# Multiple Deformation Mechanisms in Adiabatic Shear Bands of a Titanium Alloy during High Strain Rate Deformation

**DOI:** 10.3390/ma17153645

**Published:** 2024-07-24

**Authors:** Xinran Guan, Dongrong Liu, Shoujiang Qu, Guojian Cao, Hao Wang, Aihan Feng, Daolun Chen

**Affiliations:** 1 School of Materials Science and Chemical Engineering, Harbin University of Science and Technology, Harbin 150040, China; 13101618335@sina.cn; 2 School of Materials Science and Engineering, Tongji University, Shanghai 201804, China; qushoujiang@tongji.edu.cn (S.Q.); aihanfeng@tongji.edu.cn (A.F.); 3 Shanghai Key Laboratory of D&A for Metal-Functional Materials, School of Materials Science & Engineering, Tongji University, Shanghai 201804, China; 4 Key Laboratory for Light-Weight Materials, Nanjing Tech University, Nanjing 210009, China; caoguojian@njtech.edu.cn; 5 Interdisciplinary Center for Additive Manufacturing, School of Materials and Chemistry, University of Shanghai for Science and Technology, Shanghai 200093, China; haowang7@usst.edu.cn; 6 Department of Mechanical, Industrial and Mechatronics Engineering, Toronto Metropolitan University, Toronto, ON M5B 2K3, Canada

**Keywords:** adiabatic shear band, martensitic transformation, nano-twinning, dynamic recovery, severe plastic deformation

## Abstract

The occurrence of adiabatic shear bands, as an instability phenomenon, is viewed as a precursor to failure caused by instability at high strain rates. Metastable β titanium alloys are extensively utilized due to their excellent mechanical properties, which are often subjected to high strain rate loads in service conditions. Understanding and studying their adiabatic shear instability behavior is thus crucial for preventing catastrophic failure and enhancing material performance. In this study via detailed microstructural analyses in the adiabatic shear region of a Ti-10V-2Fe-3Al alloy subjected to high strain rates, it was observed that α″ martensitic transformation and nano-twinning plus β-to-α phase transformation with α″ martensite as an intermediate phase occurred, in addition to substantial fine grains. The grain refinement mechanisms were mainly related to dynamic recovery dominated by dislocation migration alongside severe plastic deformation.

## 1. Introduction

Due to their low density, high strength, and reasonable ductility/toughness at room temperature, metastable β-titanium alloys have been developed and successfully used in aircraft fuselages, wings, and landing gears where reliability and safety are of vital importance [1,2]. At the same time, the increasingly harsh environment of industrial applications places higher demands on the materials, making it inevitable that metastable β-titanium alloys will be subjected to high strain rates in service, while premature catastrophic failure of critical components must be avoided [3,4].

Adiabatic shear banding (ASB) is an important and common deformation instability phenomenon in high strain rate conditions, being often before ductile fracture [5], and has been widely studied since its discovery. The formation of ASB is explained by the “thermoplastic instability” theory, which states that the rate of thermal diffusion at high strain rates is extremely limited relative to the heat generated by plastic deformation and is close to being adiabatic. The instability phenomenon is a result of competing thermal softening, strain hardening, and strain rate hardening [6,7,8].

In the high-performance advanced engineering applications, ASB is commonly encountered. In the forging process, the occurrence of adiabatic shear is indicative of material failure and should be avoided as much as possible [6,9]. In high-speed machining processes, this phenomenon can be utilized to reduce machining temperatures and forces, thereby improving machining efficiency and surface quality [10,11]. However, in the process of projectile penetration, ASB can diminish the penetration capacity of the projectile [12]. Nonetheless, some “self-sharpening” projectile materials exploit this instability phenomenon to enhance their penetration capabilities [13]. In explosive events, materials inevitably endure high strain rate loads, reaching up to 10^6^ s^−1^, and severe plastic deformation. It is essential to prevent the occurrence of adiabatic instability in the context of armor protection [14,15]. Thus, on one hand, efforts should be made to avoid the occurrence of this plastic instability as ASB often leads to the accumulation of damage and reduction in flow stress, resulting in further concentrated deformation and eventual failure. On the other hand, this phenomenon can be utilized to enhance the efficiency of mechanical processing and improve the penetration performance of projectile materials.

Due to the extreme conditions experienced by ASB, its formation may be related to a variety of deformation mechanisms. The specific mechanisms are correlated with the type of materials [6]. Substantial grain refinement is the most common phenomenon in ASB and is crucial for the initiation and development of ASB and the change in mechanical behavior. Research by Rittle et al. [16] suggests that dynamic recrystallization (DRX) may serve as a potential cause inducing adiabatic shear instability in the early stages of deformation—a result similar to the findings of Magagnosc et al. [17]. Furthermore, Lieou et al. [5] conducted a study using polycrystalline plasticity thermodynamics theory, which indicated that DRX provides a crucial softening mechanism to explain the stress reduction in adiabatic shear instability. However, there is some controversy regarding the mechanism of grain refinement in ASB. Jiang et al. [18] studied the microstructural evolution of pure titanium ASB, revealing that the microstructure in ASB consists of ultrafine grains, with an average grain size decreasing from the initial 20 μm to 0.1–1 μm and equiaxed ultrafine grains being the product of DRX. Similarly, Li et al. [19] studied the microstructure of ultrafine-grained pure titanium ASB and observed that the initial 120 nm grain-sized structure could still undergo significant refinement in ASB, with a grain size of approximately 40 nm. These studies were accompanied by a significant temperature increase in the ASB region, enabling the possibility of DRX occurrence. However, other researchers suggest that DRX may not be completed during high-speed loading processes, and substantial grain refinement is the result of dynamic recovery (DRV). Pérez-Prado et al. [20] investigated the microstructural evolution of a Ta-W alloy ASB, observing grain refinement in the center of the ASB but with a more evident texture. Through dynamic calculations, they demonstrated that subgrain boundaries cannot be refined by rotation and relaxation, suggesting that grain refinement should be attributed to the division and breakage of initial grains by subgrain boundaries. This is consistent with the observations of Wang et al. [21] and Guan et al. [22].

To elucidate the evolution of deformation mechanisms within ASB, particularly the grain refinement mechanism, is crucial for enhancing the high strain rate service performance of materials and for achieving a rational prediction of adiabatic shear instability phenomena. Therefore, this study focused on this important issue, utilizing hat-shaped specimens and a split Hopkinson pressure bar (SHPB) to induce ASB in the designated area and employing high-resolution transmission electron microscopy (HRTEM) combined with focused ion beam (FIB) to conduct an in-depth investigation into the microstructural evolution and grain refinement mechanism in the ASB region of a metastable β-titanium alloy Ti1023. The research results in this study were aimed to advance the understanding and enrich the theory of adiabatic shear instability.

## 2. Materials and Methods

**Figure 1:**
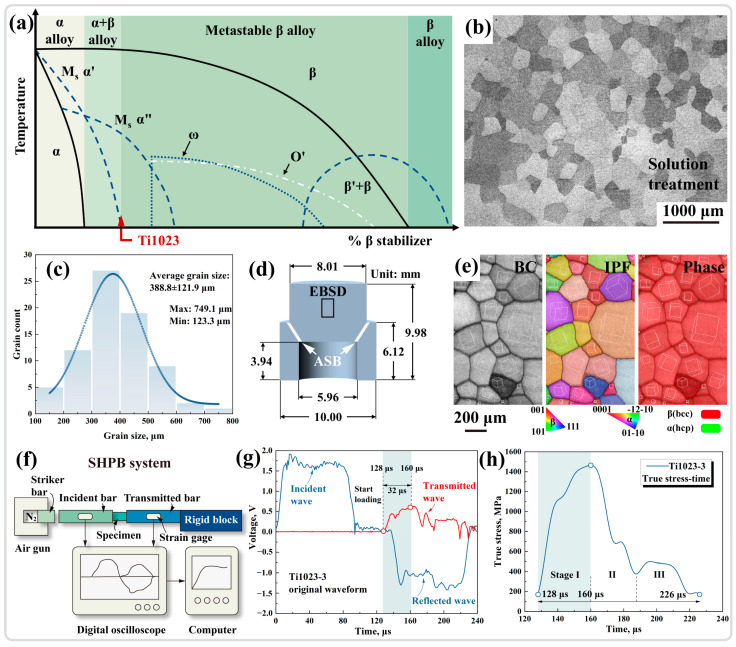
(**a**) Titanium alloy phase diagram labeling titanium alloy classification. (**b**) OM image and (**c**) grain size distribution of Ti1023 alloy after solution treatment. (**d**) Geometry and dimensions of the hat-shaped specimen. (**e**) Band contrast (BC), IPF orientation map, and phase distribution results within the hat-shaped specimen. (**f**) SHPB system schematic illustration. (**g**) Original waveforms during dynamic loading. (**h**) True stress vs. time curve.

The nominal composition of the Ti1023 alloy used in this study is Ti-10V-2Fe-3Al (wt.%). Based on the content of β-stabilizing elements (V and Fe as β-stabilizing elements and Al as an α-stabilizing element), the region of the Ti1023 alloy in the phase diagram was determined. As depicted in Figure 1a, the Ti1023 alloy is a typical metastable β titanium alloy. To avoid the interference from the multi-phase structure of the alloy in subsequent analyses, the Ti1023 alloy was subjected to a solution treatment above the β transformation temperature of 1068 K (i.e., 1103 K for 1 h, water quenching) to obtain a β single-phase microstructure. Metallographic (OM) analysis of the post-solution treatment was conducted using a Zeiss Axio Observer.5m optical microscope (Carl Zeiss AG, Jena, Germany), revealing an equiaxed and coarse β single-phase microstructure as shown in Figure 1b with an average grain size of ~389 μm (Figure 1c). The initial microstructure being β single phase was further verified through the observation of the post-solution-treated original microstructure using a Zeiss Gemini 300 scanning electron microscope (SEM) (Carl Zeiss AG, Jena, Germany) equipped with an Oxford electron backscatter diffraction (EBSD) detector (Oxford Instruments, Oxford, UK) (Figure 1e). Dynamic loading experiments were conducted on the post-solution-treated hat-shaped specimen using a split Hopkinson pressure bar (SHPB). The incident, transmitted, and reflected waves through strain gauges in the incident and transmitted bars connected to digital oscilloscopes were recorded for analyzing the strain rate, stress, strain, time, and other related parameters. The SHPB system setup is shown in Figure 1f. Further characterization and analysis of the ASB induced by forced shear in the hat-shaped specimen post-dynamic loading were performed using backscatter electron (BSE) imaging with a Zeiss Gemini 300 SEM (Carl Zeiss AG, Jena, Germany) at an accelerating voltage of 20 kV and an aperture size of 60 μm. A detailed characterization of the ASB region was conducted using a combination of FEI Helios UX FIB (FEI, Hillsboro, OR, USA) and JEM 2100F (JEOL, Tokyo, Japan) transmission electron microscopy (TEM).

## 3. Results and Discussion

### 3.1. Dynamic Mechanical Response

By analyzing the original wave of the dynamic loading process of the SHPB (Figure 1g), it can be inferred that during the continuous dynamic loading, a drop in the transmitted wave started to occur at 160 μs, indicating the generation of damage, i.e., the onset of adiabatic shear instability [4,23]. As reflected in the true stress–time curve (Figure 1h), the stress reached its peak at 160 μs, followed by a stress collapse, which was indicative of adiabatic shear instability, where the loading process started at 128 μs and ended at 226 μs with a duration of 98 μs. The entire dynamic mechanical response could be divided into three stages: The first stage comprised the elastic deformation and the double-yielding phenomenon caused by stress-induced martensitic phase transformation, the second stage was represented by the stress collapse, and the third stage was characterized by the stress dynamic equilibrium.

### 3.2. ASB Microstructural Evolution

**Figure 2:**
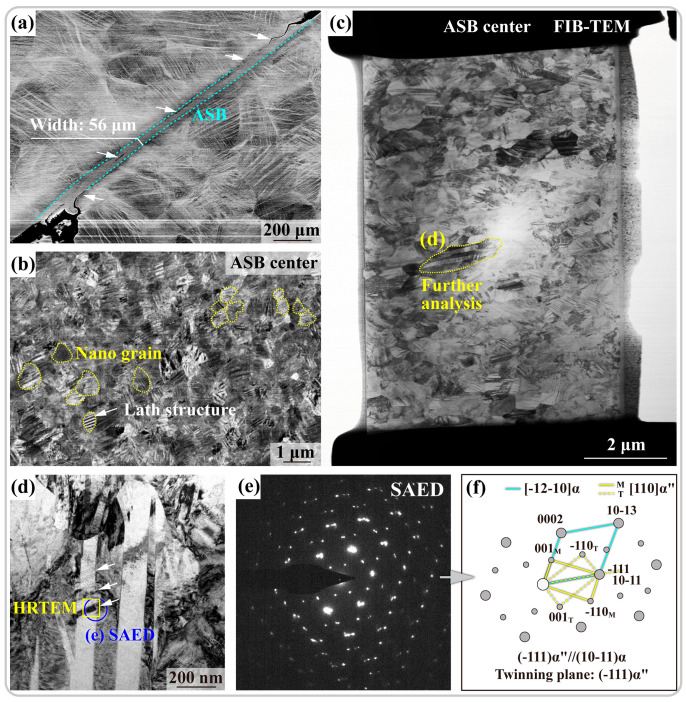
(**a**) A low-magnification BSE image containing an ASB. (**b**) ASB center at a higher magnification. (**c**) TEM BF image of ASB center. (**d**) High-magnification TEM BF of the marked area in (**c**). (**e**) SAED patterns from blue-circled area in (**d**). (**f**) Calibration results for SAED patterns in (**e**).

**Figure 3:**
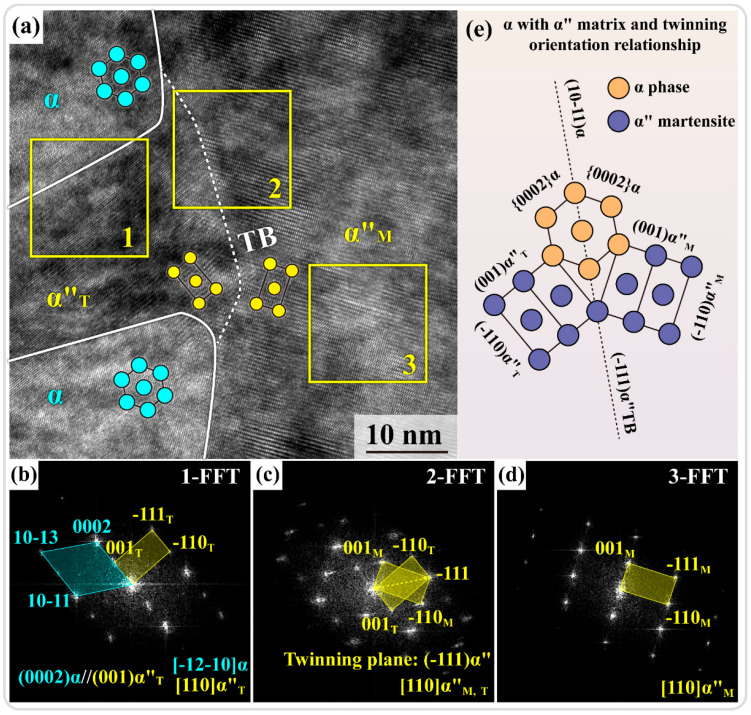
(**a**) HRTEM image in the yellow-boxed region in Figure 2d. (**b**–**d**) FFT results from areas 1–3 in (**a**), respectively. (**e**) Schematic illustration of orientation relationships between α phase and α″ matrix and twin.

An ASB of a width of ~56 μm can be seen from a low-magnification backscattered electron (BSE) image in Figure 2a, where the difference in the microstructure between the ASB and the surrounding matrix is clearly visible. Cracks and voids were also observed at the edges and inside the ASB, which could cause the catastrophic fracture at high strain rates [24,25]. High-magnification BSE analysis performed at the center of the ASB (Figure 2b) revealed that the grains in the center of the ASB were highly refined (with an average grain size of ~478 nm). As indicated by yellow outlines, a dark contrast lath structure was extensively observed within the nanograins, as indicated by white arrows. However, the relative low magnification of SEM prevented in-depth analysis, so that HRTEM combined with FIB positioning was used to characterize the fine structures of ASB more precisely. The TEM bright field (BF) results, as shown in Figure 2c, were consistent with the BSE results, pointing to widespread nanograin and lath structures in the center of the ASB. Further details of the grains indicated in Figure 2c are shown in Figure 2d, with a ~90° image rotation. The white contrast coarse laths were seen in parallel rows, within which fine dark contrast lath structures were visible. The selected area electron diffraction (SAED) analysis (Figure 2e) uncovered that three sets of diffraction spots existed in this region as {111}α″ twins and [-12-10]α, with an orientation relationship of (-111)α″//(10-11)α and [110]α″//[-12-10]α. The lattice parameter, crystal system, and the corresponding space group of the phases that may occur in the adiabatic shear region are summarized in Table 1. For further analysis of this structure, HRTEM was performed, and the results are shown in Figure 3.

Figure 3a shows the HRTEM image from the yellow box indicated in Figure 2d, with the results of the fast Fourier transform (FFT) analysis of three areas shown in Figure 3b–d. The FFT results from area 1 in Figure 3a are shown in Figure 3b, where [-12-10]α and [110]α″_T_ twins have an orientation relationship: (001)α″_T_//(0002)α. As a typical metastable β titanium alloy, the β stabilizing elements in Ti1023 enabled the attainment of a room-temperature β single-phase microstructure. However, as indicated in Figure 1a, the position of Ti1023 in the phase diagram suggested that the β stability of the alloy was still relatively limited. Thus, β phase lay in a supersaturated metastable state after solution treatment, and it had a tendency to transform to α phase under dynamic loading. However, due to the large lattice strain in the direct β-to-α transformation, it was more favorable to coordinate the β-to-α phase transformation through α″ martensite as an intermediate phase or transient phase [26]. Two types of martensite exist in titanium alloys: α′ with an HCP crystal structure and α″ with a centered orthogonal structure [27]. The choice between these two types of martensite is intricately linked to the chemical composition of titanium alloys. α′ typically forms in titanium alloys containing lower levels of β-stabilizing elements [28]. In the Ti1023 alloy investigated in the present study, the α′ martensitic transformation was hindered by its relatively high content of β-stabilizing elements [29]. FFT results further revealed the presence of {111}α″ martensite twin in area 2 (Figure 3c) and just [110]α″ martensite matrix in area 3 (Figure 3d). The location of the twin boundary (TB) is indicated as a while dashed line in Figure 3a. The orientation relationships between the α phase and the α″ matrix and twin are shown in Figure 3e. The {0002}α//(001)α″ and (-111)α″ twin boundary are parallel to (10-11)α. A combination of these HRTEM and SAED analyses allowed us to determine that the white contrast laths in Figure 2d were α, the adjacent gray contrast structure was α″ martensite, and the dark contrast {111}α″ twins were distributed within the α laths. The twin boundaries, as special high-energy grain boundaries, were more likely to act as nucleation sites for α″-to-α transformation.

### 3.3. ASB Grain Refinement

Grain refinement is a common outcome from the microstructural transformation within ASB, and the formation of ultrafine grains is mostly interpreted as a result of DRX. Temperature, as one of the key factors influencing the deformation mechanisms, also plays an important role in the occurrence of DRX in ASB. The temperature at the center of the ASB can be estimated to be ~676 K (0.38 T_m_) through the conversion of plastic work and heat, as reported in our recent study [30]. Since the conventional DRX based on the grain boundary migration mechanism is difficult to perform in high strain rate conditions, a rotational dynamic recrystallization (RDR) mechanism based on the subgrain boundary transformation along with its kinetic calculation given in Equation (1) [31] was used to analyze the grain refinement phenomenon within the ASB,

(1) $$\begin{array}{c} t=\frac{LkTf(\theta )}{4\delta \eta D_{\mathrm{bo}}\exp(-Q_{b}/RT)}\\ f(\theta )=\frac{3\tan(\theta )-2\cos(\theta )}{3-6\sin(\theta )}+\frac{2}{3}-\frac{4\sqrt{3}}{9}\ln(\frac{2+\sqrt{3}}{2-\sqrt{3}})+\frac{4\sqrt{3}}{9}\ln\frac{\tan(\theta /2)-2-\sqrt{3}}{\tan(\theta /2)-2+\sqrt{3}} \end{array}$$

**Figure 4:**
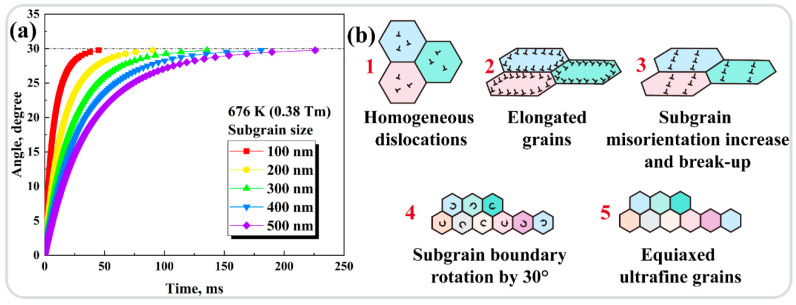
(**a**) Time required to rotate the 100–500 nm subgrain size boundaries by 30° at 676 K. (**b**) Schematic diagram illustrating the grain refinement mechanisms.

The meaning and value of the constants and variables in Equation (1) were specified in [30] and are also outlined in Table 2. The time required for subgrain sizes of 100–500 nm to complete DRX via the RDR mechanism at the estimated temperature of 676 K in the ASB center was calculated and is shown in Figure 4a. The grain refinement mechanism is shown in Figure 4b, which can be divided into the following five stages: (1) homogeneous distribution of dislocations within the initially equiaxed β-Ti grains; (2) initial deformation to generate elongated grains; (3) continued deformation with a gradual accumulation of dislocations at the subgrain boundaries and gradual subdivision of the elongated grains; (4) the subgrain boundary hindering the movement of other dislocations, causing more dislocations to accumulate at the subgrain boundary, with gradually increasing misorientations between the subgrain grains as if the elongated grains are broken to accommodate the strain; and (5) the strain energy stored at the subgrain boundaries released by rotating the subgrain boundary by 30°, causing the subgrains to relax into equiaxed ultrafine grains. Since the results of the RDR kinetic calculations shown in Figure 4a demonstrated that the subgrain boundary rotation could not be completed during the rapid dynamic loading within 0.098 ms (or 98 μs, Figure 1g,h) at the estimated peak temperature of 676 K, which was even lower than the recrystallization temperature of a highly cold-worked commercially pure titanium (919–942 K) [32], only the first four stages would occur within the ASB in this study. Thus, the grain refinement mechanism was mainly related to the DRV process by the migration of dislocations, along with the severe plastic deformation at high strain rates like equal channel angular extrusion and high-pressure torsion [33,34,35].

## 4. Conclusions

The microstructural evolution of Ti1023 ASB was characterized in detail, and the grain refinement mechanisms were discussed. The following conclusions could be drawn:

(1) Adiabatic shear instability was observed to occur during high strain rate loading, with the dynamic mechanical response exhibiting three stages: elastic deformation and stress-induced α″ martensitic transformation, followed by stress collapse and finally stress dynamic equilibrium.
(2) In addition to significant grain refinement, {111}α″ nano-twins and α laths were observed in the ASB center, and there was an orientation relationship of (001)α″//(0002)α and [110]α″//[-12-10]α between α and α″, suggesting that α″ acted as an intermediate phase to coordinate the β-to-α phase transformation.
(3) The peak temperature in the ASB center reached only ~676K; thus, the mechanism of significant grain refinement might not be DRX but rather DRV dominated by dislocation migration and severe plastic deformation under high strain rates.

## Figures and Tables

**Table 1:** Crystallographic parameters of the phases present in ASBs.

Phase	Lattice Parameter	Crystal Structure	Space Group
α phase	a = b = 0.293 nm, c = 0.466 nm	Hexagonal	P63/mmc
β phase	a = b = c = 0.325 nm	Cubic	Im-3m
α″ martensite	a = 0.301 nm, b = 0.490 nm, c = 0.463 nm	Orthorhombic	Cmcm

**Table 2:** Meaning and value of constants and variables used in Equation (1).

Constant and Variable	Meaning	Value
*L*	Average subgrain diameter	100–500 nm
*k*	Boltzmann’s constant	1.38 × 10^−23^ J·K^−1^
*T*	Absolute temperature	676 K
*δ*	Grain boundary thickness	5.8 × 10^−10^ m
*η*	Grain boundary energy	1.19 J·m^−2^
*D_bo_*	Constant related to grain boundary diffusion	2.8 × 10^−5^ m^2^·s^−1^
*Q*	Activation energy for grain boundary diffusion	312 kJ·mol^−1^
*θ*	Subgrain misorientation	0–30°
*R*	Gas constant	8.314 J·mol^−1^·K^−1^

## Data Availability

The raw/processed data required to reproduce these findings cannot be shared at this time as the data also form part of an ongoing study.
